# Effect of 20 mph speed limits on traffic injuries in Edinburgh, UK: a natural experiment and modelling study

**DOI:** 10.1136/jech-2023-221612

**Published:** 2024-05-07

**Authors:** Kyriaki (Kelly) Kokka, Glenna Nightingale, Andrew James Williams, Ali Abbas, Valentin Popov, Stephen Sharp, Ruth F Hunter, Ruth Jepson, James Woodcock

**Affiliations:** 1 MRC Epidemiology Unit, University of Cambridge, Cambridge, UK; 2 Scottish Collaboration for Public Health Research and Policy, University of Edinburgh, Edinburgh, UK; 3 University of St Andrews, St Andrews, UK; 4 Centre for Public Health, Queen's University Belfast, Belfast, UK

**Keywords:** ACCIDENTS, PUBLIC HEALTH, EPIDEMIOLOGY

## Abstract

**Introduction:**

There is limited research evaluating 20 mph speed limit interventions, and long-term assessments are seldom conducted either globally or within the UK. This study evaluated the impact of the phased 20 mph speed limit implementation on road traffic collisions and casualties in the City of Edinburgh, UK over approximately 3 years post implementation.

**Methods:**

We used four sets of complementary analyses for collision and casualty rates. First, we compared rates for road segments changing to 20 mph against those at 30 mph. Second, we compared rates for the seven implementation zones in the city against paired control zones. Third, we investigated citywide casualty rate trends using generalised additive model. Finally, we used simulation modelling to predict casualty rate changes based on changes in observed speeds.

**Results:**

We found a 10% (95% CI −19% to 0%) greater reduction in casualties (8% for collisions) for streets that changed to 20 mph compared with those staying at 30 mph. However, the reduction was similar, 8% (95% CI −22% to 5%) for casualties (10% collisions), in streets that were already at 20 mph. In the implementation zones, we found a 20% (95% CI −22% to −8%) citywide reduction in casualties (22% for collisions) compared with control zones; this compared with a predicted 10% (95% CI −18% to −2%) reduction in injuries based on the changes in speed and traffic volume. Citywide casualties dropped 17% (95% CI 13% to 22%) 3 years post implementation, accounting for trend.

**Conclusion:**

Our results indicate that the introduction of 20 mph limits resulted in a reduction in collisions and casualties 3 years post implementation. However, the effect exceeded expectations from changes in speed alone, possibly due to a wider network effect.

**WHAT IS ALREADY KNOWN ON THIS TOPIC**

- There is limited evidence regarding the effectiveness of 20 mph speed limit interventions, with previous studies presenting varying findings and rarely evaluating long-term outcomes.

**WHAT THIS STUDY ADDS**

- Three analyses (at city, area and road link level) all suggested that citywide 20 mph speed limit intervention resulted in the reduction of road traffic collisions and casualties.

**HOW THIS STUDY MIGHT AFFECT RESEARCH, PRACTICE OR POLICY**

- Policymakers should consider implementing citywide 20 mph speed limit interventions as part of a package of measures, to reduce collisions and casualties.

## Introduction

Road traffic collisions are a worldwide public health problem; they are among the eight leading causes of death globally resulting in approximately 1.3 million preventable deaths annually and leave between 20 and 50 million people with non-fatal preventable injuries.^1^ In 2019, the UK reported 1752 road traffic-related fatalities^2^; 161 of which were in Scotland^3^ a relatively low and decreasing number locally and nationally but not tolerated under the UK Vision Zero and sustainable safety approach to end fatal and serious traffic injuries.

Traffic speed is a key risk factor in road traffic collisions and injuries severity.^1^ There is a consistent link between higher speeds and a higher number of traffic collisions, injuries severity and their frequency because increasing speed decreases the field of vision to notice road changes and increases the stopping distance for the driver to react timely. Reducing speed is a key part of reducing the risk of road traffic collisions and the number of casualties.^4^ Bellefleur and Gagnon’s^5^ literature review found that traffic calming measures reduced traffic casualties. Taylor *et al* 6 found that lowering speed in urban roads has great potential for collision reduction. Thus, a simple public health approach to match travel speed and road safety in urban environments is via the speed limit. An umbrella review^7^ found that 20 mph policies are associated with collision and injury reductions. The primary reason for implementing such schemes is to reduce traffic casualties but they often have broader influences. Jones and Brunt^8^ provided a literature review of the evidence on the effect of 20 mph policies not only on traffic casualties but also on air quality and active travel. The most recent review at global scale^9^ included a range of public health outcomes such ascollisions, casualties, mode of transport, noise pollution, air quality, inequalities and liveability (eg, physical activity and perceptions of safety).

Many countries have implemented 20 mph (32 kph) speed calming policies (such as speed limits or zones) to prevent deaths and injuries. Such schemes are becoming popular around the world and within the UK and have been adopted by various local governments to reduce road traffic collisions and casualties and to improve liveability.^9–11^ City-wide 20 mph speed limits were introduced in Portsmouth^12^ and Bristol,^13^ while 20 mph speed zones were implemented in Hull and London.^14^ Other local authorities have introduced 20 mph restrictions on a smaller, more localised scale on a pilot basis (see Cleland *et al* 9 for a summary).

The City of Edinburgh Council has a long-standing policy of introducing 20 mph speed limits, initially focused on residential areas and around schools that counted for 50% of the city’s roads being at 20 mph. In January 2015, a citywide 20 mph speed limit network for Edinburgh was approved by the Transport and Environment Committee. It aimed to extend the 20 mph speed limits to the city centre, main shopping streets and residential areas (80% of the city’s roads) while retaining a network of roads at 30 mph and 40 mph in the city suburbs.

This is the second in a series of papers, it incorporates results from the first paper on the evaluation of the traffic speed and volume in the City of Edinburgh before and after the speed limit implementation.^15^ The overall aim of the second paper is to investigate the impact of the 20 mph speed limits on both road traffic collisions (the event) and casualties (people) and their severity (slight, serious, fatal).

### Objectives

1. Evaluate the impact of 20 mph speed limit casualties between streets that changed to 20 mph and streets that stayed at 30 mph in the City of Edinburgh.
2. Evaluate differences in 20 mph implementation zones and matched control zones from other cities of Scotland.
3. Investigate the overall trend for casualties.
4. Employ simulation modelling in severities.

## Methods and data

### Intervention

The 20 mph speed limit interventions were introduced between 31 July 2016 and 5 March 2018 in seven implementation zones in the City of Edinburgh. The implementation was in a phased fashion (or stepped-wedge design)^16^ such that some implementation zones were still at 30 mph while others were already at 20 mph (see online supplemental materials 1 and 2). This is a complex intervention with policy, signage, education and enforcement components but it does not involve any physical traffic calming measures such as speed humps.

10.1136/jech-2023-221612.supp1 Supplementary data

Collisions referred to the incidents and casualties were the people injured in the collisions, meaning that one collision could result in more casualties. Thus, both variables were examined in the pre-20 mph and post-20 mph period.

### Data and Geographic Information Systems manipulations

We matched the implementation zones with Data Zones, a key small area statistics geography in Scotland, using the Scottish Index of Multiple Deprivation (SIMD) 2016. For each implementation zone, we sought a matching control zone elsewhere in Scotland by using administrative geographies that clustered Data Zones of similar sizes. Given Scotland’s small size, the list of potential matches was limited. The number of possible matches for each implementation zone is in online supplemental material 3. The matching process considered seven separate domains of SIMD, a six-category urban-rural classification, and area population density, employing ranks for SIMD domains, frequencies, mean and median as well as histograms to identify the most similar match for urban-rural classification. It was 1:1 and the final selection of a matching geography for the implementation areas was a manual process.

The shapefiles with the road speed limit network and the seven implementation zones were linked with STATS19; the dataset of police recorded road traffic collisions in the UK. The final dataset contained the STATS19 variables enhanced with the Geographic Information Systems (GIS) variables such as the ‘zone information’ and the ‘speed limit segment’. These variables indicated whether a given collision occurred in an implementation or control zone (and if so, which one), and in which speed limit segment category.

First, we located collisions in a specific road segment that indicated at which speed limit category this collision occurred. Using STATS19 eastings and northings we acquired the location of the accidents. We matched the collisions with the closest road segment in 12 m distance (12 m was selected as it gave the most accurate results in visual inspection of test sites). We conducted an inspection of the junction locations for which it was challenging to identify their closest road (because multiple road segments may meet). More details on the nearest line method are in online supplemental material 4. Second, we locate collisions within one of the implementation and control zones by finding the area which every spatial collision overlapped with one of the identified zones.

The data manipulations and GIS analysis were done using the statistical software, R (V.4.2.2; R Core Team 2022). The code is available online (https://github.com/20mph-study).

### Methods

We undertook four types of analysis, using the data at street, zone and city level. First, we compared the roads that remained at 30 mph versus those that changed to 20 mph. Second, we compare changes in collisions and casualties in the control and implementation zone and between them. Third, we analysed the impact of the intervention on trends in city wide casualties. Finally, we used simulation modelling to predict changes in causalities (by severity) based on changes in speed and volume.

#### Road segments (objective 1)

We calculated annual rates of collisions and casualties and collisions for the road segment categories in the pre-20 mph and post-20 mph period (3 years pre and 1.83 years post). Rates were sums of collisions or casualties divided by total time. The road segment categories inside the implementation zones of Edinburgh are categorised as (i) existing 20 mph streets before the speed limit implementation, (ii) streets that were 30 mph before and after the speed limit implementation and (iii) streets that were 30 mph before the speed limits, but 20 mph after (local 20 mph and main 20 mph). To calculate the impact of interventions, we compared rates of collisions and casualties between roads that remained 30 mph to those that changed to 20 mph (from 30 mph).

$$diff\;rates=\frac{rate_{post}-rate_{pre}}{rate_{pre}}\times 100$$

$$Diff\;\%diff=\%diff\;rates_{20\;mph}-\%diff\;rates_{30\;mph}$$

#### Implementation-control zones (objective 2)

Similarly, we calculated the annual rates and percentage difference in rates of collisions and casualties in the pre-20 mph and post-20 mph period, comparing implementation and control zone pairs. The implementation periods differ between zones in the post-20 mph period (as described in online supplemental materials 5–8) and the timeframes considered for the rate calculations differ from that used for the road segment calculations. The formulas were the same as those used in the road segments. We compare control with implementation zones by producing a difference in differences.

$$Diff\;\%diff=\%diff\;rates_{implementation}-\%diff\;rates_{control}$$

#### Overall road traffic casualties—time series modelling (objective 3)

We employed a generalised additive model (GAM) to estimate the trends in road traffic casualties at the city level. The fitted values from the GAM were used to generate estimates of the trends for road traffic casualties, pre-intervention and post-intervention. The model incorporates secular trend and seasonality, but also the intervention effect. These were all cast as smooth (thin plate splines) functions. An offset was used to account for the difference in the number of days per month. We had 273 monthly observations, 30 of which were after the intervention. A sub-model was constructed for data up to the first date of intervention and then casualties were predicted from the following month till the last data-date. The predicted data were compared with that observed during the same time period. We followed the GAM as described in Popov *et al* 17 using the mgcv package.^18^

#### Collisions and casualties (objective 4)

We used Elvik’s exponential models^19^ to simulate the changes in casualties based on the speed and volume changes. Elvik developed a simpler version, using speed categories, of Hauer and Bonneson’s exponential functions according to which the effects of a given change in speed depends on initial speed. The estimates produced by Elvik were based on aggregated data before and after the speed change. We used the values for the speed range of 20 mph–30 mph for three injury categories (fatal, serious, injury). Because the modelling is at the collision level, we defined the severity of each collision as that of the most severely injured casualty. We compared the trends to the predictions of casualty annual rates made by Elvik’s model.

#### Exponential model

$$\begin{array}{l} {pred\;inj}_{\left(speed\right)}=inj\;rate\;bef\\ \times e^{(mean\;speed\;after-mean\;speed\;bef)\times estimate\;based\;on\;accident\;severity} \end{array}$$

#### Speed and volume combined (linear)

$${pred\;inj}_{\left(speed+vol\right)}={pred\;inj}_{\left(speed\right)}\times \left(1+\frac{volume\;after-volume\;bef}{volume\;bef}\right)$$

## Results

### Road segments (objective 1)

We found a 42% reduction in collision rates in 20 mph road segments (main and local streets combined) and 44% reduction for existing 20 mph streets. In streets that stayed at 30 mph collision rates fell by 35%, giving a difference in difference of 8% (95% CI −2% to 18%). For causalities, we found the largest percentage reduction (43%) in casualty rates for streets switched to 20 mph (main and local streets combined). Road segments that stayed at 30 mph exhibited the lowest percentage reduction in casualty rates (table 1), with a difference in difference of 10% (95% CI 0% to 19%).

**Table 1:** Average annual road traffic casualty and collision rates by speed limit road segments citywide

Road type	Sum (pre)	Sum (post)	Rate (pre)	Rate (post)	Rate (diff)	Rate (% diff)	Diff in diff (%)
Collisions							
20 mph existing streets	354	121	118 (98 to 141)	66 (51 to 84)	−52 (−69 to –35)	−44 (−57 to –33)	−10 (−24 to 5)
20 mph local and main streets	1568	550	523 (479 to 570)	301 (268 to 337)	−222 (−258 to –186)	−42 (−48 to –37)	−8 (−18 to 2)
30 mph	802	321	267 (236 to 301)	175 (150 to 203)	−92 (−119 to –65)	−35 (−43 to –26)	REF
Casualties							
20 mph existing streets	389	139	130 (108 to 154)	76 (59 to 95)	−54 (−702 to –36)	−42 (−53 to –30)	−8 (−22 to 6)
20 mph local and main streets	1757	613	585 (539 to 634)	335 (300 to 373)	−250 (−288 to –212)	−43 (−48 to –38)	−10(−19 to 0)
30 mph	934	378	311 (277 to 348)	207 (180 to 237)	−104 (−133 to –75)	−33 (−41 to –26)	REF

The rates (counts/total years) and difference in rates are accompanied by 95% CI using the Poisson distribution and the delta method.

### Implementation—control zones comparison (objective 2)

Both the casualty and collision rates fell across the seven implementation zones and across all the zones except zone 4 (table 2). There was a greater reduction in rates in all the implementation zones compared with control zone for casualties and all but one (1b) zone for collisions.

**Table 2:** Comparison of collision rates—implementation versus control zones with 95% CI (Poisson distribution and delta method)

Zone	Rate pre (20 mph zones)	Rate post (20 mph zones)	Rate pre (matched control)	Rate post (matched control)	Rate: % diff_20 mph_	Rate: % diff_Control_	Diff in diff (%)
Collisions							
1a	156 (132 to 182)	115 (95 to 138)	24 (15 to 36)	18 (11 to 28)	−26 (−36 to –16)	−25 (−51 to 1)	−1 (−29 to –59)
1b	21 (13 to 32)	19 (11 to 30)	15 (8 to 25)	13 (6 to 22)	−10 (−41 to 21)	−13(−49 to 23)	3 (−44 to 51)
3	284 (252 to 319)	189 (163 to 218)	117 (97 to 140)	96 (78 to 117)	−34 (−40 to –26)	−18 (−31 to –5)	−16 (−30 to –1)
4	63 (48 to 81)	43 (31 to 58)	71 (55 to 90)	75 (59 to 94)	−32 (−48 to –16)	6 (−15 to 26)	−38 (−63 to −11)
5	89 (71 to 110)	48 (35 to 64)	254 (224 to 287)	220 (191 to 251)	−46 (−57 to –35)	−13 (−23 to –4)	−33 (−47 to −18)
6	69 (54 to 87)	39 (28 to 53)	106 (87 to 128)	97 (79 to 118)	−44 (−58 to –28)	−9 (−25 to 8)	−35 (−58 to –12)
Total	897 (839 to 958)	593 (546 to 643)	587 (540 to 636)	519 (475 to 656)	−34 (−41 to 27)	−12 (−29 to 5)	−22 (−40 to –2)
Casualties							
1a	170 (145 to 198)	125 (104 to 149)	26 (17 to 38)	20 (12 to 31)	−27 (−36 to –17)	−23 (−48 to 2)	−4 (−30 to 23)
1b	25 (16 to 37)	22 (14 to 33)	16 (9 to 26)	15 (8 to 25)	−12 (−40 to 16)	−6 (−43 to 31)	−6 (−52 to 41)
3	323 (289 to 360)	222 (194 to 253)	144 (121 to 169)	112 (92 to 135)	−31 (−38 to –24)	−22 (−34 to –11)	−9 (−22 to 4)
4	70 (55 to 88)	48 (35 to 64)	88 (71 to 108)	95 (77 to 116)	−31 (−47 to –16)	8 (−11 to 27)	−39 (−63 to –15)
5	103 (84 to 1250)	57 (43 to 74)	303 (270 to 339)	248 (218 to 281)	−45 (−56 to –34)	−18 (−26 to –10)	−27 (−40 to –13)
6	78 (62 to 97)	47 (35 to 63)	126 (105 to 150)	120 (99 to 143)	−40 (−55 to –25)	−5 (−21 to 11)	−35 (−57 to –13)
Total	1014 (953 to 1078)	680 (630 to 733)	703 (652 to 757)	610 (563 to 660)	−33 (−40 to –26)	−13 (−23 to –4)	−20 (−22 to –8)

### Overall road traffic casualties—time series modelling (objective 3)

**Figure 1:**
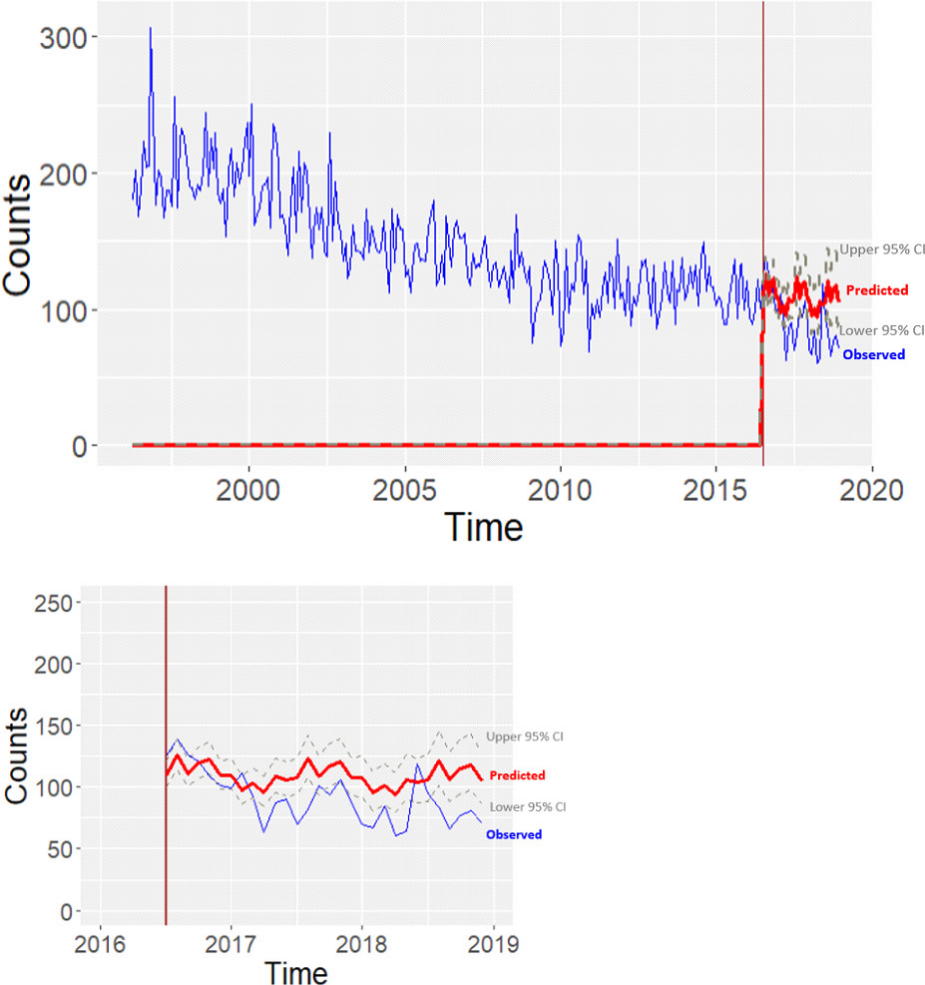
Visualisation of the casualties trends post-20 mph for the observed data and predicted data with a zoom in the post-20 mph period. The red line is the predicted and the blue line observed. The grey lines are the lower and upper 95% CI for the predicted trend.

Figure 1 shows a downward trend of traffic casualties in the pre implementation and post implementation period and a zoomed in in the post implementation period. The blue line indicates the observed data and then a sub-model was constructed to predict what the number of casualties would have been if the intervention was not implemented (with grey lines 95% CIs). The predicted data were compared with that observed during the same post-period. Based on this trend we would have expected a post implementation annual rate of 1306 (95% CI 1261 to 1352) casualties. However, in figure 1 we note that there is a steepening of the declining trend during the months closely following the intervention, with the observed trend line mostly outside of the CI for the predicted casualties. This results in an observed post implementation annual rate of 1079 (95% CI 1037 to 1120) casualties, giving a difference of 228 (95% CI 166 to 289) casualties per year, 17% (95% CI 13 to 22) difference between predicted and observed casualty rates (table 3). Overall, the key finding is that the effect of the intervention is to further decrease the number of casualties. Details on the model specification and fit are in online supplemental materials 9–13.

**Table 3:** Comparison of the monthly and annual rates with 95% CI rates based on Poisson distribution for road traffic casualties for the predicted and observed data pre-20 mph and post-20 mph (all figures have been rounded), normal approximation for CIs in difference of rates and delta method for the difference CIs

Timeframe	Annual rates
Post-20 mph trend (2.42 years)	
Predicted from 31 July 2016 to December 2018	1306 (1261 to 1352)
Observed from 31 July 2016 to December 2018	1079 (1037 to 1120)
Difference between predicted and observed	228 (166 to 289)
Difference between predicted and observed (%)	17 (13 to 22)
Pre-20 mph trend (3 years)	
Observed from 31 July 2013 to 30 July 2016	1414 (1341 to 1490)
3 years pre vs 2.42 years post	
Difference between observed pre and post	−332 (−391 to –273)
Difference between observed pre and post (%)	−23 (−27 to –20)

### Collisions and casualties: simulation modelling based on Elvik model (objective 4)

For the implementation zones speed fell by an average of 1.3 mph (23.63 vs 22.29), while traffic volumes fell by 2% (3641 vs 3555), for full speed and volume data for each zone (online supplemental material 14).

When we compared modelled (based on speed and volume) estimates of changes in injury rates to those observed (table 4), the observed rates were smaller, with the two being closest for serious injuries. Fatalities, as rare events, are expected to vary considerably from year. In the summer of 2019, Scottish police changed the reporting method of collision severities. Before June 2019 many serious injuries were reported as slight. This had an impact on the number of slight and serious injury numbers. Data for 6 months of the post implementation period affected by this change.

**Table 4:** Observed and modelled changes in injury rates by severity with 95% CI based on Poisson distribution for counts and SEs from Elvik

Accident severity	Exponent	Observed annual rates			Exponential modelled rates	Difference between predicted post and observed pre-20 mph (%)	Difference between predicted and observed post-20 mph (%)	
	Estimate	Pre-20 mph	Post-20 mph	% diff	Predicted_(speed+vol)_	% diff	diff	% diff
Injury	0.034 (0.014 to 0.054)	897 (839 to 958)	593 (546 to 642)	−31 (−38 to –24)	817 (762 to 875)	−9 (−17 to 0)	224 (150 to 298)	27 (20 to 35)
Serious	0.069 (0.045 to 0.093)	119 (99 to 142)	107 (99 to 142)	−10 (−34 to 14)	101 (82 to 123)	−15 (−38 to 8)	−6 (−36 to 24)	−6 (−36 to 24)
Fatal	0.069 (0.045 to 0.093)	5.7 (2 to 13)	4.7 (2 to 13)	−18 (−143 to 108)	4.9 (1.6 to 11.7)	−14 (−135 to 107)	0.2 (−7 to 8)	4 (−145 to 154)
All casualties	–	1022 (960 to 1087)	705 (654 to 759)	−31 (−38 to –24)	923 (864 to 985)	−10 (−18 to –2)	218 (138 to 298)	24 (16 to 31)

The casualty rates by severity are based on the ‘Accident Severity’ variable in the collisions dataset and averaged over implementation zone (online supplemental material 14).

## Discussion

### Key findings

In summary, our findings show significant reductions in road traffic casualties and collisions following the 3-year implementation of 20 mph speed limit intervention in Edinburgh.

If we begin by examining the results at the city level and then progress to the smallest geographies. At the city level, there was a further steepening of the declining secular trend (objective 3) of road traffic casualties, with an additional 17% faster reduction in casualties. The implementation zones exhibited larger reductions in road traffic casualty rates than that for the control (objective 2), with an overall reduction of around 20% (95% CI −22% to −8%). This compares with what we would have expected from changes in speeds and volume of around a 10% reduction in casualties (objective 4), using Elvik’s model. The evidence was least clear for the specific road segments (objective 1). The rates fell on all road types. They fell more on roads that changed to 20 mph faster than those that stayed at 30 mph and roads that stayed at 20 mph showed a similar decline to those that changed to 20 mph.

Overall these results show that injuries fell rapidly in Edinburgh during the period of the study compared with the previous already downward trend and compared with controlled areas. The reduction was greater than we would expect from changes in speeds on volume alone. The effect was as pronounced on streets that stayed at 20 mph as those that changed to 20 mph but was greater than on streets that stayed at 30 mph.

### Strengths and limitations

This study is based on a natural experiment. The major strength of this study is the use of multiple controls to allow triangulation of our results. A second strength is that we mapped collisions with speed limits based on the road network instead of relying on STATS19 speed records. Third, this study is among very few studies of 20 mph speed limit interventions that assessed data 3 years after the implementation.

Limitations include our use of STATS19 data for collisions and casualties, which will underestimate collisions and to a lesser extent injuries. Second, we did not have data on active travel, and changes to walking and cycling rates are a potential confounder.

Considering first the city level analyses. There was a change in drink-driving laws in Scotland during this period, which might have contributed to the large overall citywide reduction in injuries beyond the previous trend. However, this should not explain the substantial reduction in the implementation zones compared with the controls. Potentially there may be other policies specific to Edinburgh that led to a faster reduction in collisions and casualties compared with other cities in Scotland. But we are not aware of other such policies that would be expected to have that affect.

For the street segments, the fact that the largest reduction in collisions was in the street segments that had already changed to 20 mph suggests that the intervention might take some time to take full effect. It is also possible there are wider area effects in which lower speeds (and perhaps more care) on a 20 mph street leads to lower speeds on other streets and that the more streets are covered the greater the behaviour change. As we do not have data on speed on street segments, we could not investigate this. Potentially pedestrians and cyclists could have diverted from streets that stayed at 30 mph to those that changed to 20 mph, which would lead to underestimating the effects.

### Compare/contrast with the literature

Although 20 mph schemes are popular worldwide, there are limited studies evaluating the impact of 20 mph interventions and particularly for casualties and thus our findings add to the limited knowledge of the effectiveness of such policies. To our knowledge, this is one of the first studies for casualties and one of few for collisions.^13 20^

A study of 20 mph speed limits in Portsmouth^21^ found that the road casualty rate fell by 22% (21% for collisions) in the 2 years after the implementation of 20 mph speed limits compared with the 3 years beforehand in the streets that changed to 20 mph. However, this study did not adjust for trends or have a control. This reduction is similar to what we observed, 20% reduction in casualties (22% in collisions).

A Department of Transport research study of the 20 mph speed limit implementation in Bristol^22^ reported reductions in annual rates of fatal, serious and slight injuries after the implementation. The range of the before period is between 6 and 7.75 years and the after period from 1.25 to 3 years. However, the study did not account for the overall trend or clearly report their methods.

A study conducted in Belfast^23^ did not show the same scale of impact on collisions and casualties in 1 year pre-implementation and 3 years post implementation likely due to the scale of the implementation; only roads in the city centre changed to 20 mph compared with the broad Edinburgh implementation of seven zones. Also, Belfast did not have any previous 20 mph limits unlike Edinburgh’s gradual implementation and also a lack of enforcement and awareness campaigns.

### Meaning of the study findings

Potential explanations for the results are (i) that the benefit of 20 mph carries on beyond the initial change (evidenced by the reduction in roads already at 20 mph), (ii) that either the 20 mph policy or other changes specific to the city (not in the control groups) led to a more rapid reduction in collisions and injuries through a mechanism beyond that of a small speed reduction on the roads that changed (eg, greater care by drivers).

### Recommendations for future policy and practice

The results of this study add to the limited evidence on the impact of 20 mph speed limit interventions. Our analysis showed reduction in casualties and casualty severities. These results indicate that 20 mph speed limits are an effective part of the toolkit to reduce collisions and casualties in the City of Edinburgh and could enforce the implementation of speed limit policies. Speed limit policies are easier to implement and be maintained, and a budget friendly intervention compared with the expensive implementation of the 20 mph zones which require enforcing measures such as speed humps, chicanes, road narrowing or planting.

The vision of a city should always be at the core of any intervention. Councils could use speed limit policies as part of an overall long-term strategy for the city to reduce road injuries by making one change at a time until the desired change will happen by itself through the combination of these small interventions. The 20 mph speed limit intervention in the city of Edinburgh is an example of such gradual implementation. In the first phase 50% of streets changed, in the second phase around 80% of the streets converted to 20 mph in the city centre and the results indicate that the scheme worked well as there is a clear sign of reduction in collisions and casualties 3 years post implementation. This complex effort to reduce casualties and improve liveability could be enhanced by other traffic calming measures and a more extended 20 mph speed limit intervention.

## Conclusion

Our findings support the case that city wide 20 mph intervention led to a reduction of road traffic collisions and casualties. These benefits were substantial and seemed to be larger than would be expected just from the reduction in speeds immediately after implementation.

## Data Availability

Data are available upon reasonable request. Stats19 analysed in this study are available via archive (https://www.data.gov.uk/dataset/cb7ae6f0-4be6-4935-9277-47e5ce24a11f/road-safety-data), and aggregated annual data are described in the summary tables in the article or included within the supplementary materials. GIS files are available upon reasonable request.

## References

[1] World Health Organization . Save lives: a road safety technical package. 2017.

[2] Department for transport, the road safety statement 2019, a lifetime of road safety moving Britain ahead.

[3] The Scottish government, road safety data report. 2019.

[4] Elvik R . The power model of the relationship between speed and road safety, update and new analyses. Oslo: Institute of Transport Economics, 2009.

[5] Bellefleur Olivier , Gagnon François . Urban traffic calming and health: a literature review. National Collaborating Centre for Healthy Public Policy, 2012.

[6] Taylor MC , Lynam D , Baruya A . The effects of drivers' speed on the frequency of road accidents. Transport Research Laboratory Crowthorne, 2000.

[7] Cairns J , Warren J , Garthwaite K , et al . Go slow: an umbrella review of the effects of 20 mph zones and limits on health and health inequalities. J Public Health 2015;37:515–20. 10.1093/pubmed/fdu067 25266281

[8] Jones SJ , Brunt H . Twenty miles per hour speed limits: A sustainable solution to public health problems in Wales. J Epidemiol Community Health 2017;71:699–706. 10.1136/jech-2016-208859 28341623

[9] Cleland CL , McComb K , Kee F , et al . Effects of 20 mph interventions on a range of public health outcomes: a meta-narrative evidence synthesis. Journal of Transport & Health 2020;17:100633. 10.1016/j.jth.2019.100633

[10] Tapp A , Nancarrow C , Davis A . Support and compliance with 20mph speed limits in great Britain. Transportation Research Part F: Traffic Psychology and Behaviour 2015;31:36–53. 10.1016/j.trf.2015.03.002

[11] Toy S , Tapp A , Musselwhite C , et al . Can social marketing make 20mph the new norm? J Transp Health 2014;1:165–73. 10.1016/j.jth.2014.05.003

[12] Atkins and M. Maher . 20mph research study: process and impact evaluation: technical report. 2018.

[13] Bornioli A , Bray I , Pilkington P , et al . The effectiveness of a 20 mph speed limit intervention on vehicle speeds in Bristol, UK: a non-randomised stepped wedge design. J Transp Health 2018;11:47–55. 10.1016/j.jth.2018.09.009

[14] Grundy C , Steinbach R , Edwards P , et al . Effect of 20 mph traffic speed zones on road injuries in London, 1986-2006: controlled interrupted time series analysis. BMJ 2009;339:b4469. 10.1136/bmj.b4469 20007666 PMC2791801

[15] Nightingale GF , Williams AJ , Hunter RF , et al . Evaluating the citywide Edinburgh 20mph speed limit intervention effects on traffic speed and volume: A pre-post observational evaluation. PLoS ONE 2021;16:e0261383. 10.1371/journal.pone.0261383 34972123 PMC8719778

[16] National Institute for Health Research (2017) . Is 20 plenty for health? Evaluation of the 20mph speed limit networks in Edinburgh and Belfast on a range of public health outcomes,

[17] Popov V , Nightingale G , Williams AJ , et al . Trend shifts in road traffic collisions: an application of hidden Markov models and generalised additive models to assess the impact of the 20 mph speed limit policy in Edinburgh. Environment and Planning B: Urban Analytics and City Science 2021;48:2590–606. 10.1177/2399808320985524

[18] Wood SN . Generalized additive models. In: Generalized additive models: an introduction with R. CRC press, 2017 Available. https://www.taylorfrancis.com/books/9781498728348

[19] Elvik R . A re-Parameterisation of the power model of the relationship between the speed of traffic and the number of accidents and accident victims [Pages 854-860]. Accid Anal Prev 2013;50:854–60:S0001-4575(12)00266-7. 10.1016/j.aap.2012.07.012 22840212

[20] Atkins . Interim evaluation of the implementation of 20 mph speed limits in Portsmouth. 2010.

[21] Department for Transport . Interim evaluation of the implementation of 20 mph speed limits in Portsmouth. 2010.

[22] Pilkington P , et al . The Bristol twenty miles per hour limit evaluation (Brite) study. 2018.

[23] Hunter RF , Cleland CL , Busby J . Investigating the impact of a 20 miles per hour speed limit intervention on road traffic collisions, casualties. J Epidemiol Community Health 2023;77:17–25. 10.1136/jech-2022-219729 PMC9763225 36379715

