# Supplementary material for: Effect of 20 mph speed limits on traffic injuries in Edinburgh, UK: a natural experiment and modelling study
Source: J Epidemiol Community Health. 2024 May 7;78(7):437–43. doi: 10.1136/jech-2023-221612 (PMC11187370; doi:10.1136/jech-2023-221612)
Supplement: Supplementary data [file jech-2023-221612supp001.pdf]

Supplementary file 1: Road network of Edinburgh

Supplementary file 1: Map of the speed limit segments: 20mph existing (20mph before and after), local and main 20mph (30mph before and 20mph after), 30mph (before and after)

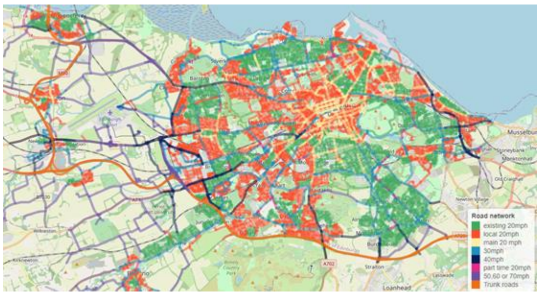

Supplementary file 2: The implementation zones

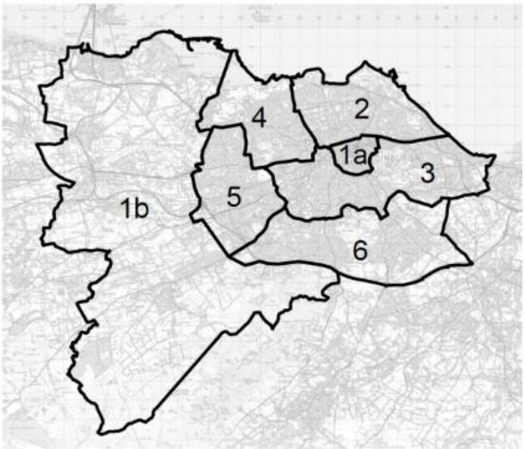

Supplementary file 3: Choosing control zones

Reviewing the larger administrative geographies in Scotland with the most similar number of Data Zones and urban rural classification to each intervention zone were selected as 'long-lists' of potential control zones. Within these long lists for each zone the population density and domains of SIMD16 were compared to identifying the most suitable control zone. Using this method, we were not able to identify a suitable control zone for implementation zone 2 (North). Finally, each control zone was translated back to Data Zones to be used across the various outcomes.

The full list of factors considered in the matching were the seven separate domains of SIMD, the 6 category urban-rural classification and the area population density. For the domains of SIMD, the ranks were used. Subsequently, apart from urban rural classification, where frequencies were used, we looked as mean and median as well as histograms to identify the most similar match, it was 1:1. With all these complications and the challenge to find a matching geography for the implementation areas the methods were simple and thus the final selections came down to a manual process.

Implementation zones and the number of possible matches

Implementation zone	1A City centre	1B	2	3	4	5	6
Number of areas to find a match from	14	16	3	2	7	10	10

Implementation-control zone pairings for the 20mph speed limit program in the City of Edinburgh. The last column with maps of the control zones shows the exact datazones selected from every city based on SIMD, urban-rural classification and the area population density.

Implementation zones	Overall SIMD 2016 rank(median)	Area	Implementation Phase	Operative Date	Matched area	Maps of control areas
1a	3490	City Centre	1	31 July 2016	Electoral ward George St/Harbour, Aberdeen	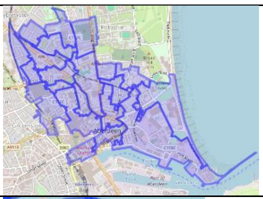
1b	5739	Rural West	1	31 July 2016	Electoral wards Tay Bridgehead and St Andrews	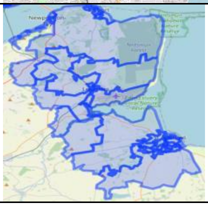
2	4473	North	2	28 February 2017	***No suitable zone found***	-
3	5630	South Central/East	2	28 February 2017	Community Health Partnership Dundee	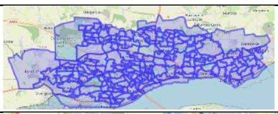
4	6103	North West	3	16 August 2017	Electoral wards Renfrew North (S13002750), Renfrew South & Gallowhill and Paisley North West	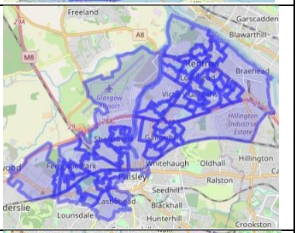
5	3562	West	3	16 August 2017	Scottish Parliament Constituency Glasgow Kelvin	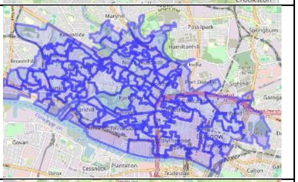
6	4828	South	4	5 March 2018	Scottish Parliament Constituency Paisley	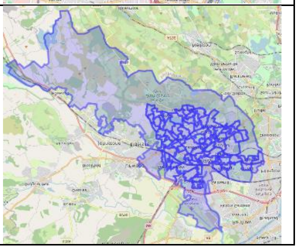

Supplementary file 4: GIS manipulation: Explanation of correcting the nearest line method in junctions

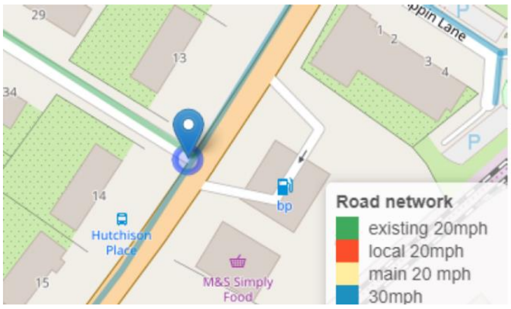

1st class road: A

GIS road trajectory before reassign: 20mph existing

GIS road trajectory after reassign: 30mph

We looked in junctions where multiple road segments may be joined (and selected the two closest road segments from the collision's location). For these injuries we looked up their road class information (from the STATS19 1st road class variable). This variable describes the class of the road that the accident happened with values A, B, C or unclassified and it is the most direct connection STATS19 offers for identifying the exact location. The closest match tended not to "find" the major roads at junctions. We investigated the bias of the closest match in junctions by comparing the proportion of major roads (1st road class = A class) with minor roads (road segment = "20mph existing streets" & "20mph local streets"). We corrected these injuries by reassigning them to the major closest road segment.

Supplementary file 5: Average annual casualty and collision rates in the city of Edinburgh by implementation zone. The rates and difference in rates are accompanied by 95% Confidence Intervals. Years pre for all zones: 3 years; years post: zone 1a and 1b [3.42 years], 2 and 3 [2.83 years], 4 and 5 [2.63 years], and 6 [1.83 years]

Zone	Sum (pre)	Sum (post)	Rate (pre)	Rate (post)	Rate (diff)	Rate (% diff)
**Collisions**						
1a	469	395	156(132,182)	115(95,138)	-41(-73,-9)	-26(-36, -16)
1b	64	66	21(13,32)	19(11,30)	-2(-9,5)	-10(-41, 21)
2	645	396	215(187,246)	140(118,165)	-75(-97,-53)	-35(-43, -27)
3	851	536	284(252,319)	189(163,218)	-95(-120,-70)	-34(-40, -26)
4	188	114	63(48,81)	43(31,58)	-20(-32,-8)	-32(-48, -16)
5	268	125	89(71,110)	48(35,64)	-41(-55,-27)	-46(-57, -35)
6	206	72	69(54,87)	39(28,53)	-30(-43,-17)	-44(-58, -28)
total	2691	1704	897(839,958)	593(546,643)	-304(-380, -228)	-34(-41, 27)
**Casualties**						
1a	511	426	170(145,198)	125(104,149)	-45(-64,-26)	-23(-48,2)
1b	76	76	25(16,37)	22(14,33)	-3(-11,5)	-6(-43,31)
2	735	450	245(215,278)	159(135,186)	-86(-109,-63)	-35(-43, -27)

Zone	Sum (pre)	Sum (post)	Rate (pre)	Rate (post)	Rate (diff)	Rate (% diff)
3	968	629	323(289,360)	222(194,253)	-101(-128,-74)	-22(-34,-11)
4	211	126	70(55,88)	48(35,64)	-22(-35,-9)	8(-11,27)
5	309	150	103(84,1250	57(43,74)	-46(-61,-31)	-18(-26,-10)
6	235	86	78(62,97)	47(35,63)	-31(-45,-17)	-5(-21,11)
total	3045	1943	1014(953,1078)	680(630,733)	-334(-415, -253)	-13(-23, -4)

Supplementary file 6: Average annual casualty rates per Control zone. The rates and difference in rates are accompanied by 95% Confidence Intervals. Years pre for all zones: 3 years; years post: zone 1a and 1b [3.42 years], 2 and 3 [2.83 years], 4 and 5 [2.63 years], and 6 [1.83 years]

Zone	Sum (pre)	Sum (post)	Rate (pre)	Rate (post)	Rate (diff)	Rate (% diff)
**Collisions**						
1a	71	42	24(15,36)	18(11,28)	-6(-13,1)	-25(-51, 1)
1b	45	30	15(8,25)	13(6,22)	-2(-8,4)	-13(-49, 23)
3	351	168	117(97,140)	96(78,117)	-21(-38, -4)	-18(-31,-5)
4	214	97	71(55,90)	75(59,94)	4(-10,18)	6(-15, 26)
5	762	286	254(224,287)	220(191,251)	-34(-59,-9)	-13(-23, -4)
6	317	73	106(87,128)	97(79,118)	-9(-27,9)	-9(-25, 8)
total	1760	696	587(540,636)	519(475,656)	-68(-133,-3)	-12(-29, 5)
**Casualties**						
1a	78	47	26(17,38)	20(12,31)	-6(-13,1)	-4(-30, 23)
1b	48	34	16(9,26)	15(8,25)	-1(-7,5)	-6(-52, 41)
3	432	196	144(121,169)	112(92,135)	-32(-50,-14)	-9(-22, 4)
4	263	124	88(71,108)	95(77,116)	7 (-9,23)	-39(-63, -15)*
5	908	322	303(270,339)	248(218,281)	-55(-82,-28)	-27(-40, -13)*
6	379	90	126(105,150)	120(99,143)	-6 (-26,14)	-35(-57, -13)
total	2108	813	703(652,757)	610(563,660)	-93(-164,-22)	-20(-22, -8)

Supplementary File 7: Comparison of collision rates – implementation vs control zones with 95% CI (Poisson distribution and delta method). Years pre for all zones: 3 years; years post: zone 1a and 1b [3.42 years], 2 and 3 [2.83 years], 4 and 5 [2.63 years], and 6 [1.83 years]

**Zone**	**Rate pre**	**Rate post**	**Rate pre**	**Rate post**	**Rate: $diff_{20mph}$**	**Rate: $diff_{Control}$**	**Rate: % $diff_{20mph}$**	**Rate: % $diff_{Control}$**	**Diff in diff (%)**
	20mph zones		Matched control		$rate_{pre} - rate_{post}$		$\frac{rate_{post}-rate_{pre}}{rate_{pre}} * 100$		$\%diff_{20mph}$ - $\%diff_{Control}$
**Collisions**									
1a	156(132,182)	115(95,138)	24(15,36)	18(11,28)	-41(-73, -9)	-6(-13,1)	-26(-36, -16)	-25(-51, 1)	-1(-29, -59)
1b	21(13,32)	19(11,30)	15(8,25)	13(6,22)	-2(-9,5)	-2(-8,4)	-10(-41, 21)	-13(-49, 23)	3(-44, 51)
3	284(252,319)	189(163, 218)	117(97,140)	96(78,117)	-95(-120, -70)	-21(-38, -4)	-34(-40, -26)	-18(-31, -5)	-16(-30, -1)
4	63(48,81)	43(31,58)	71(55,90)	75(59,94)	-20(-32, -8)	4(-10,18)	-32(-48, -16)	6(-15, 26)	-38(-63, -11)
5	89(71,110)	48(35,64)	254(224, 287)	220(191, 251)	-41(-55, -27)	-34(-59, -9)	-46(-57, -35)	-13(-23, -4)	-33(-47, -18)
6	69(54,87)	39(28,53)	106(87,128)	97(79,118)	-30(-43, -17)	-9(-27,9)	-44(-58, -28)	-9(-25, 8)	-35(-58, -12)
total	897(839,958)	593(546, 643)	587(540, 636)	519(475, 656)	-304(-381, -227)	-68(-170, 34)	-34(-41, 27)	-12(-29, 5)	-22(-40, -2)
**Casualties**									
1a	170(145,198)	125(104, 149)	26(17,38)	20(12,31)	-45(-64, -26)	-6(-13,1)	-27(-36, -17)	-23(-48,2)	-4(-30, 23)
1b	25(16,37)	22(14,33)	16(9,26)	15(8,25)	-3(-11,5)	-1(-7,5)	-12(-40,16)	-6(-43,31)	-6(-52, 41)
3	323(289,360)	222(194, 253)	144(121, 169)	112(92,135)	-101(-128, -74)	-32(-50, -14)	-31(-38, -24)	-22(-34, -11)	-9(-22, 4)
4	70(55,88)	48(35,64)	88(71,108)	95(77,116)	-22(-35, -9)	7 (-9,23)	-31(-47, -16)	8(-11,27)	-39(-63, -15)
5	103(84,1250	57(43,74)	303(270, 339)	248(218, 281)	-46(-61, -31)	-55(-82, -28)	-45(-56, -34)	-18(-26, -10)	-27(-40, -13)
6	78(62,97)	47(35,63)	126(105, 150)	120(99,143)	-31(-45, -17)	-6 (-26,14)	-40(-55, -25)	-5(-21,11)	-35(-57, -13)
total	1014(953, 1078)	680(630, 733)	703(652, 757)	610(563, 660)	-334(-415, -253)	-93(-164, -22)	-33(-40, -26)	-13(-23, -4)	-20(-22, -8)

Supplementary file 8:

Basic analysis comparing the mean number of casualties and collisions before and after the 20mph limit change in Edinburgh. The data before the limit changes relate to the period August 2013 through July 2016, while the after data relate to the period March 2018 to December 2019. Figures in **bold** are statistically significant differences ($p<0.05$) according to a two-sample t-test with unequal variances.

(a) Difference in mean number of casualties after limit change compared to before

		All months	Jan**	Feb**	Mar	Apr	May	Jun	Jul	Aug	Sep	Oct	Nov	Dec
All Edinburgh	20mph existing streets	**-4.49**	-6.67	-10.00	-3.83	-0.50	-6.50	-7.33	-4.83	-6.33	-2.67	-3.00	-5.00	-1.00
	**20mph local streets**	**-5.04**	-5.67	-4.33	-0.17	-1.17	-2.50	-3.50	-3.83	-5.00	-5.67	**-10.17**	-10.50	-8.33
	**20mph main streets**	**-15.87**	-9.67	-9.00	**-15.67**	-15.83	**-26.00**	-13.17	-16.17	-12.00	-13.83	-9.50	**-13.83**	**-34.83**
	30mph	**-8.78**	-10.00	-10.67	-8.50	-7.00	-10.83	-10.33	**-14.67**	**-8.67**	-2.33	**-10.33**	-5.50	-9.67
Zones 1a, 1b, 3, 4, 5, 6*	20mph existing streets	**-3.43**	-8.33	-5.67	-1.50	-0.83	-4.83	**-7.00**	-5.33	-3.67	-2.83	-1.67	-3.50	0.50
	**20mph local streets**	**-4.53**	-6.00	-3.00	-3.83	-0.50	-1.67	-2.00	-5.00	-4.17	-3.83	-4.33	**-9.50**	-10.83
	**20mph main streets**	**-11.36**	-8.33	-10.33	-11.17	-10.00	**-20.00**	-8.17	-10.17	-12.67	-13.00	-2.50	-9.83	-22.33
	30mph	**-7.27**	-10.33	-9.67	-9.00	-6.83	-3.00	-8.67	-8.83	**-9.50**	-0.17	-8.50	-6.67	-8.00
	Control 20mph**	**2.24**	-	-	0.33	1.67	2.67	1.67	0.00	5.00	1.00	2.67	2.33	4.67
	Control 30mph**	**-20.29**	-	-	-32.00	-5.33	-16.00	-12.33	-25.00	-25.00	-17.00	-21.67	-12.00	-27.67

*As it was not possible to identify a control zone for Zone 2, these rows combine the other intervention zones and their matched control zones
** Only one post intervention observation so unable to undertake T-test

(b) Difference in mean number of collisions after limit change compared to before

		All months	Jan **	Feb **	Mar	Apr	May	Jun	Jul	Aug	Sep	Oct	Nov	Dec
All Edinburgh	20mph existing streets	**-4.33**	-9.00	-6.33	-4.83	-1.00	-6.00	-5.50	-4.50	-6.67	-3.00	-3.33	-5.00	-1.33
	**20mph local streets**	**-4.30**	-6.00	-3.33	-2.50	-0.17	-0.83	**-5.00**	**-4.67**	-3.33	-3.83	**-8.17**	**-9.00**	-6.67
	**20mph main streets**	**-14.28**	-8.00	-9.00	**-12.33**	-13.33	**-21.67**	-14.83	-10.00	-11.50	-12.50	-14.00	-10.67	**-31.50**
	30mph	**-7.60**	-4.33	-8.33	-6.00	-4.83	-9.83	-10.33	**-11.50**	-10.33	-1.33	**-8.67**	-5.50	-10.83
Zones 1a, 1b, 3, 4, 5, 6*	20mph existing streets	**-3.37**	-7.67	-2.67	-2.50	-1.33	-4.67	**-5.83**	-5.00	-4.33	-3.17	-2.67	-3.50	0.17
	**20mph local streets**	**-3.67**	-5.33	-2.67	-4.00	0.17	0.00	-3.00	**-4.83**	-2.50	-3.00	**-3.67**	-8.00	-8.17
	**20mph main streets**	**-10.49**	-7.67	-10.33	**-8.17**	-8.17	**-16.33**	-10.17	-8.33	-11.00	-11.17	-7.17	-8.50	**-21.00**
	30mph	**-6.31**	-5.00	-7.67	-6.67	-5.33	-3.33	-9.50	-8.17	-10.00	1.00	**-6.67**	-5.67	-9.17
	Control 20mph **	**2.15**	-	-	0.33	0.67	2.00	1.67	0.67	5.00	1.00	2.67	2.67	4.67
	Control 30mph **	**-15.47**	-	-	-24.33	-6.00	-9.67	-9.67	-17.00	-18.00	-14.33	-19.67	-10.00	-18.67

*As it was not possible to identify a control zone for Zone 2, these rows combine the other intervention zones and their matched control zones
** Only one post intervention observation so unable to undertake T-test

Supplementary file 9: GAM model specification

Let X_t be a random variable for the casualties such as that $X_t \sim NB(\mu_t, n)$ and

$$\log(\mu_t) = \log(days_t) + f_{tr}(t) + f_{seas}(s)$$

BMJ Publishing Group Limited (BMJ) disclaims all liability and responsibility arising from any reliance placed on this supplemental material which has been supplied by the author(s)

with t = 1, …, 216 and s = 1, …, 12. $\log(days_t)$ is an offset term, f_{tr} and f_{seas} are smooth functions with cubic spline basis for the trend and for the seasonality features.

With the effect of the intervention, the mean equation is modified:

$\log(\mu_t) = \log(days_t) + f_{tr,1}(t) + f_{seas}(s) + d_t f_{tr,2}(t)$, with t = 1, …, 216 and s = 1, …, 12 , $f_{tr,1}$ gives the effect of the trend without the intervention, $f_{tr,2}$ give the additional effect of the intervention on the trend and a dummy variable $d_t = \begin{cases} 0, & t = 1,2,\ldots,243 \\ 1, & otherwise \end{cases}$.

Supplementary file 10: GAM model fit

The model fit indicates high significance for the smooth terms related to the trend, seasonality, and the additional impact on the trend (all three p-values < 0.001). This reinforces the statistical significance of the intervention's effect. The visual representation in Supplementary file 13 displays the three smooths. The GAM accurately captures the declining trend, including the observed 'trough' in later years. The residual randomization test and diagnostic plots (Supplementary file 11) indicate no under-smoothing in the three smooths (p-values > 10%). Plots against the linear predictor and responses against fitted values show no discernible patterns. The ACF and partial ACF of residuals in Supplementary file 12 suggest successful mitigation of autocorrelation, affirming the overall reasonableness of the model fit.

Supplementary file 11: Basic model checking plots for the GAM with the intervention.

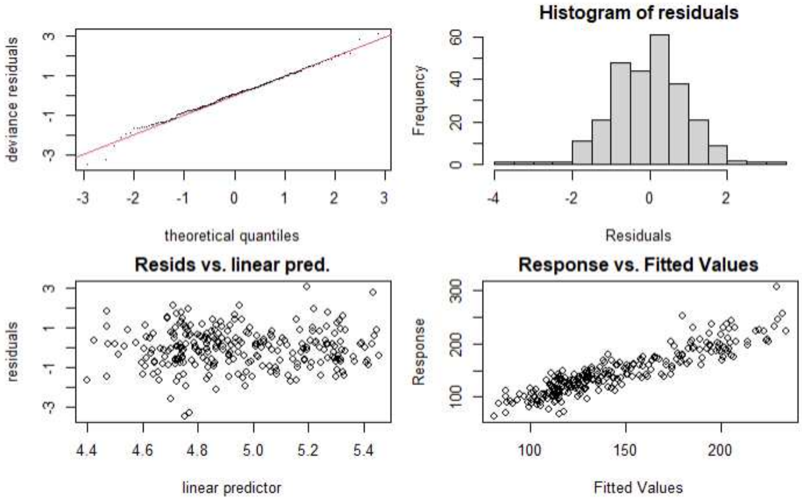

Supplementary file 12: ACF and partial ACF residual plots for the GAM with intervention.

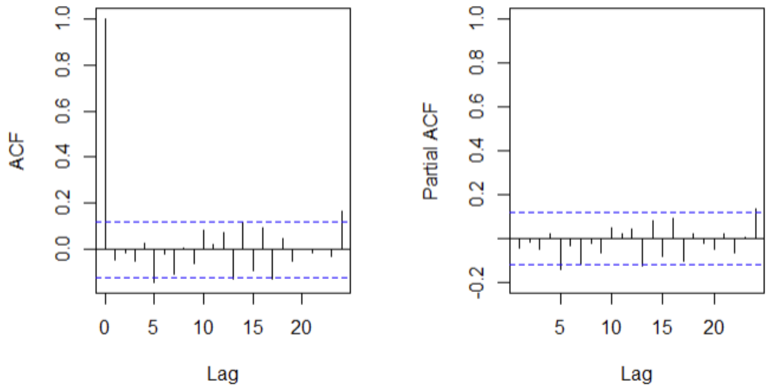

Supplementary file 13: Fitted smooths. Upper plot gives the smooth for the trend, lower left plot gives the smooth for the seasonality, lower right plot gives the smooth for the additional effect of the intervention.

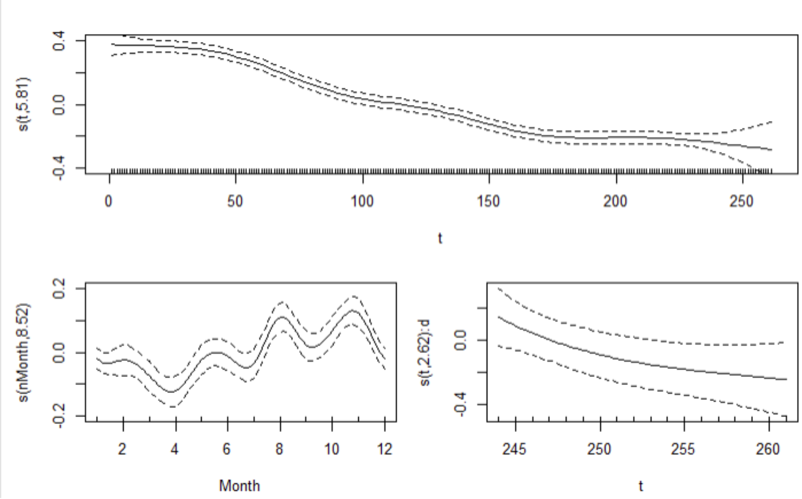

Supplementary file 14: Variables and data used in Elvik modelling

Zone	Speed pre	Speed post	Diff in speed	Years pre	Years post	Volume pre	Volume post	Collision severity rates pre			Collision severity rates post		
								Injury	Serious	Fatal	Injury	Serious	Fatal
1a	24.14	22.07	2.07	3	3.42	2822	2847	156	24	0.7	115	22	0.6
1b	23.84	21.43	2.41	3	3.42	5572	5321	21	2	0.5	19	4	0
2	23.53	22.2	1.33	3	2.83	4423	4279	215	29	1.6	140	26	1.4
3	25.4	23.89	1.51	3	2.83	4169	3962	283	36	1.3	189	29	1.4
4	26.54	25.75	0.79	3	2.63	3035	2954	63	9	0.4	43	8	0
5	20.14	18.97	1.17	3	2.63	2163	2415	89	10	0.6	48	11	0.8
6	20.25	20.66	-0.41	3	1.83	1690	1569	69	10	0.5	39	7	0.5
all	23.63	22.29	1.34			3641	3555	897	119	5.7	593	107	4.7
